# Using a consumer-based wearable activity tracker for physical activity goal setting and measuring steps in pregnant women with gestational diabetes mellitus: exploring acceptance and validity

**DOI:** 10.1186/s12884-021-03900-8

**Published:** 2021-06-08

**Authors:** Samantha F. Ehrlich, Jill M. Maples, Cristina S. Barroso, Kathleen C. Brown, David R. Bassett, Nikki B. Zite, Kimberly B. Fortner

**Affiliations:** 1 Department of Public Health, The University of Tennessee, Knoxville, 390 HPER, 1914 Andy Holt Ave, Knoxville, TN 37996 USA; 2 The University of Tennessee, Graduate School of Medicine, 1924 Alcoa Highway, Knoxville, TN 37920 USA; 3 College of Nursing, The University of Tennessee, Knoxville, 1200 Volunteer Blvd, Knoxville, TN 37996 USA; 4 Department of Kinesiology, Recreation, and Sport Studies, The University of Tennessee, Knoxville, 1914 Andy Holt Ave, Knoxville, TN 37996 USA

**Keywords:** Pregnancy, Physical activity, Walking, Steps, Gestational diabetes

## Abstract

**Background:**

Activity monitoring devices may be used to facilitate goal-setting, self-monitoring, and feedback towards a step-based physical activity (PA) goal. This study examined the performance of the wrist-worn Fitbit Charge 3™ (FC3) and sought opinions on walking and stepping-in-place from women with gestational diabetes (GDM).

**Methods:**

Participants completed six 2-min metronome-assisted over ground bouts that varied by cadence (67, 84, or 100 steps per minute) and mode (walking or stepping-in-place; *N* = 15), with the sequence randomized. Steps were estimated by FC3 and measured, in duplicate, by direct observation (hand-tally device, criterion). Equivalence testing by the two one-sided tests (TOST) method assessed agreement within ± 15%. Mean absolute percent error (MAPE) of steps were compared to 10%, the accuracy standard of the Consumer Technology Association (CTA)™. A subset (*n* = 10) completed a timed, 200-m self-paced walk to assess natural walking pace and cadence. All participants completed semi-structured interviews, which were transcribed and analyzed using descriptive and interpretive coding.

**Results:**

Mean age was 27.0 years (SD 4.2), prepregnancy BMI 29.4 kg/m^2^ (8.3), and gestational age 32.8 weeks (SD 2.6). The FC3 was equivalent to hand-tally for bouts of metronome-assisted walking and stepping-in-place at 84 and 100 steps per minute (i.e., *P* < .05), although walking at 100 steps per minute (*P* = .01) was no longer equivalent upon adjustment for multiple comparisons (i.e., at *P* < .007). The FC3 was equivalent to hand-tally during the 200-m walk (i.e., *P* < .001), in which mean pace was 68.2 m per minute (SD 10.7), or 2.5 miles per hour, and mean cadence 108.5 steps per minute (SD 6.5). For walking at 84 and 100 steps per minute, stepping-in-place at 100 steps per minute, and the 200-m walk, MAPE was within 10%, the accuracy standard of the CTA™. Interviews revealed motivation for PA, that stepping-in-place was an acceptable alternative to walking, and competing responsibilities made it difficult to find time for PA.

**Conclusions:**

The FC3 appears to be a valid step counter during the third trimester, particularly when walking or stepping-in-place at or close to women’s preferred cadence.

## Background

Gestational Diabetes Mellitus (GDM), defined as glucose intolerance first recognized during pregnancy [1], is diagnosed at 24 to 28 weeks gestation and affects approximately 6% of pregnancies in the U.S. [2]. Lifestyle behavior change, i.e., diet and physical activity (PA), is a key component of GDM management, as 70–85% of women with GDM are able to manage the condition with lifestyle behavior change alone [3]. The American Diabetes Association’s *Standards of Medical Care in Diabetes* (*Standards*) recommend that after a GDM diagnosis, treatment begin with medical nutrition therapy, PA, and, depending on pregestational weight class, weight management [3]. The *Standards* provide guidance on medical nutrition therapy (i.e., an individualized nutrition plan) and weight management [i.e., weight gain according to the 2009 Institute of Medicine recommendations [4], which are specific to women’s prepregnancy body mass index (BMI) category, recommending 12.5–18 kg, 11.5–16 kg, 7–11.5 kg, and 5–9 kg for underweight women, healthy weight women, women with overweight and women with obesity, respectively], but offer no further advice pertaining to PA [3].

The American College of Obstetricians and Gynecologists (ACOG) suggests that women who were physically active before pregnancy or habitually participate in vigorous-intensity activity continue these activities during pregnancy [5]. ACOG also recommends 150 min per week of moderate-intensity aerobic exercise, and that walking for 10–15 min after each meal can improve glycemic control [5]. Although randomized controlled trials of exercise interventions in women with GDM clearly demonstrate improvements in glucose [5, 6], behavioral PA interventions that are appropriate for the clinical setting are largely lacking [7].

Successful behavioral interventions for weight management (i.e., lifestyle counseling on diet and PA) in pregnant and postpartum women have relied upon goal setting, self-monitoring, and feedback on progress towards health behavior goals [8–10]. Walking is the most commonly reported mode of PA in pregnant women in the U.S. [11]. Consumer-based wearable activity trackers have gained popularity in recent years and may be used to facilitate goal-setting, self-monitoring, and feedback, and ultimately help to increase walking and overall step count in pregnant women [12]. Indeed, meta-analyses report that PA interventions utilizing consumer-based wearable activity trackers significantly increase steps per day [13].

In a lab-based study of non-pregnant adults, stepping-in-place while watching TV commercials had similar caloric requirement to walking at 3.0 mph on a treadmill [14], thus stepping-in-place may serve as an alternative, convenient mode of moderate-intensity activity. A small randomized controlled trial of sedentary, overweight adults (non-pregnant) found that stepping-in-place or walking around the room during TV commercials induced similar, favorable increases in daily step count to walking, and that both stepping-in-place and walking induced similar decreases in percent body fat and waist and hip circumference as compared to controls [15]. Stepping-in-place can be also accurately recorded by pedometer [15].

The present study sought to: 1) assess the criterion-related validity of the Fitbit Charge 3™’s step count for walking and stepping-in-place during the third trimester of pregnancy in women with GDM, and estimate their natural walking cadence during this period, and 2) examine women’s thoughts and feelings on a step-based PA goal (e.g., encouraging women to step-in-place when unable to walk) through semi-structured interviews.

## Methods

The study was approved by the University of Tennessee, Knoxville, IRB (UTK IRB-19–04,989-FB), and all methods carried out in accordance with relevant guidelines and regulations. Participants were recruited from the University of Tennessee Medical Center Knoxville (UTMC), a regional referral academic medical center serving a 21 county area in East Tennessee. Women receiving prenatal care from practices affiliated with UTMC who receive a diagnosis of GDM are referred to the Maternal–Fetal Medicine (MFM) practice for GDM management. UTMC delivered 4,710 infants in 2019, of which 447 (9.5%) were diagnosed with GDM. Eligible women with GDM receiving care from MFM were identified by clinic staff and referred to study personnel.

Eligibility criteria included: proficiency in English (i.e., no translator needed), no prior diagnoses of diabetes outside of pregnancy, prepregnancy BMI < 45 kg/m^2^, non-smoker, diagnosed with GDM during the index pregnancy by published criteria [5, 16] and prescribed medical nutrition therapy for GDM treatment at their first appointment. In this setting, the first appointment for GDM typically entails diabetes education, a baseline dietary assessment and development of a GDM meal plan. This is followed by approximately 1 week of self-monitoring and tracking dietary intake and capillary glucose values, which are then reviewed with a diabetes educator at a follow up appointment.

Study personnel reviewed the medical records of women referred from clinic staff for eligibility. Study physicians (NZ and KF) additionally ensured that women were free of absolute contraindications to physical activity during pregnancy [17] and provided final approval for recruitment/participation. Study personnel then met approved, eligible women at their next MFM appointment to distribute information about the study and invite them to participate. Study visits were scheduled with interested, eligible women who were < 36 weeks gestation at the time of recruitment.

Study visits took place on the University of Tennessee, Knoxville, main campus. Participants received a $50 Walmart gift card to compensate for their time completing the study visit and those that elected to complete the timed 200-m walk received an additional $5. At the study visits, written informed consent was first obtained and a short survey was completed to collect demographic and health information. The survey assessed PA prior to pregnancy and during the last 30 days with the Stanford Leisure-Time Activity Categorical Item (L-Cat), for which participants assign themselves to 1 of 6 descriptive categories ranging from inactive to very active [18, 19]. Participants then completed a timed, 200-m self-paced walk (on a track) to assess natural walking cadence, as data pertaining to natural walking cadence in late pregnancy are limited [20]. The participants were instructed to walk briskly yet comfortably. The self-paced walk was optional since the track was not guaranteed to be available during all study visits (e.g., weather), and thus performed by a sub-set of participants. Steps taken during the self-paced walk were estimated by a wrist-worn Fitbit Charge 3™ (hereafter, ‘FC3’) and determined, in duplicate, by direct observation and using a hand-tally device.

A FC3 was set up for each participant prior to their visit (i.e., with date of birth and current weight). It was worn on the non-dominant wrist as they completed 2-min bouts of metronome-assisted PA that varied by cadence (i.e., 67, 84, or 100 steps per minute) and by mode (i.e., walking or stepping-in-place), thus 6 bouts total; bout order was randomized and participants rested, as needed, between the bouts. The cadences 67, 84, and 100 steps per minute were selected because 100 steps per minute is a reasonable threshold indicative of moderate intensity walking in non-pregnant adults [21], and there is some evidence to suggest that women walk more slowly in late pregnancy as compared to early pregnancy and the non-pregnant state [20, 22]. The FC3’s exercise function for ‘Walking’ was utilized to capture the device’s step count during walking bouts and the ‘Treadmill’ function to capture the step count for the stepping-in-place bouts. The FC3 exercise functions allow users to manually record and track bouts of PA, by mode, in an ‘Activity Log’ that is visible online and via a cell phone app [7]. Use of these functions would allow users and researchers to differentiate between tracked episodes of walking versus stepping.

For the walking bouts, participants walked down a long, wide, temperature-controlled hallway while letting their hands swing naturally at their sides and made wide turns (i.e., arc-shaped) to change direction. For the stepping bouts, they were instructed to lift their feet to a height of 6 inches (as marked on the wall) and to let their hands swing naturally at their sides. The metronome used to help participants stay on cadence was carried by study staff, who walked or stepped alongside the participants to help them maintain the appropriate cadence and guide them through the wide turns. Actual steps were again determined, in duplicate, by direct observation using a hand-tally device, and averaged.

The final portion of the study visit was the semi-structured interview. Interviews were conducted by trained research staff in a private room and audio recorded. Questions asked about participants’ thoughts and feelings on PA, i.e., opportunities to engage in PA during a typical day, fitting 30 min of PA into a typical day, challenges that accompany PA during late pregnancy, encouraging women to step-in-place when they are unable to walk (e.g., due to bad weather or other circumstances), and using a FC3 to record and track episodes of walking and stepping.

### Analyses

Quantitative analyses were conducted in SAS v9.3. Mean preferred walking cadence and pace were estimated from the time and step counts obtained during the 200-m walk. The number of steps recorded by the FC3 during each bout was divided by the number of hand-tallied steps to determine the percentage of hand tallied steps recorded by the FC3. Mean absolute percentage error (MAPE) was also calculated [i.e., Σ (|(hand-tallied step count—Fitbit Charge 3 step count)/(hand-tallied step count)|× 100)] [23] and compared to the accuracy standard of the Consumer Technology Association (CTA)™ (i.e., within 10%) [24]. Equivalence testing evaluated agreement between the FC3 and hand-tallied step counts. Specifically, the two one-sided tests (TOST) method was used to test the null hypothesis that the step count sources are not equivalent, using a ± 15% equivalence region [23, 25]. A Bonferroni correction was applied to adjust for multiple comparisons (i.e., 7 total, *P* < 0.007). Bland Altman plots provided visual assessment of bias and agreement. For the Bland Altman plots, distributions of the differences were assessed for normality with Kolmogorov–Smirnov tests and visually by histogram plots; mean differences (bias) and their 95% confidence intervals, and limits of agreement (LOA) are also presented.

Audio recordings of the semi-structured interviews were transcribed using InqScribe (https://www.inqscribe.com/) software and cross-checked to ensure accuracy. A inductive thematic analysis approach was used to identify themes that emerged from the data [26]. Codification of transcripts was iterative and collaborative. Two investigators (CB and KB) manually and independently reviewed and coded one transcript. Then the investigators met to discuss the provisional codes and corresponding rationale. After the first meeting, the two investigators continued to independently code the remaining transcripts. Throughout this iterative and collaborative process, the two investigators held two additional consensus meetings to discuss their codes (agreements and discrepancies of assigned codes) and compare how they applied the codes to the data. Differences in coding were resolved and documented during these consensus meetings to finalize the descriptive and interpretive coding.

## Results

**Fig. 1:**
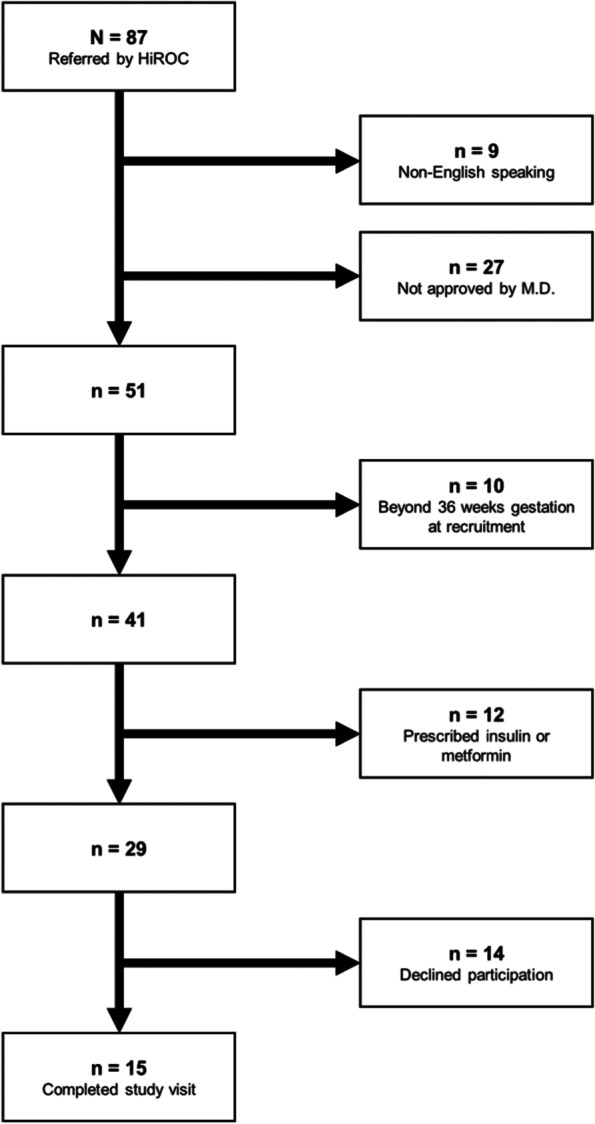
Recruitment flow chart

In June, July, and August 2019, 87 women were referred to study staff; 29 of them met eligibility criteria and received physician approval to contact. Of these 29 women, 15 completed study visits (Fig. 1). Ten of the 15 elected to participate in the optional timed 200-m walk; environmental concerns (e.g., sun exposure, heat) were the primary reason for opting out of the 200-m walk.

Mean gestational age at the study visit was 32.8 weeks (SD 2.6). On average, participants were 27.0 years of age (SD 4.2) and self-reported prepregnancy BMI was 29.4 kg/m^2^ (8.3). In regards to PA prior to pregnancy, 73.3% reported PA below recommended levels (i.e., a minimum of 30 min, 5 times per week). For PA during the last 30 days, 93.3% reported PA below recommended levels (i.e., all but one participant). Participant characteristics are presented in Table 1. Forty percent were primiparas and 80% were married or living with their partners. One-third participated in the Special Supplemental Nutrition Program for Women, Infants, and Children (WIC) and 40% were insured by state Medicaid (i.e., TennCare).

**Table 1:** Participant characteristics (*N* = 15)

	**n (%)**
**Age** (years)	
18–20	1 (6.7)
21–25	6 (40.0)
26–30	4 (26.7)
31–35	4 (26.7)
**Parity**	
0	6 (40.0)
1	4 (26.7)
2 +	5 (33.3)
**Prepregnancy BMI**	
≤ 20	3 (20.0)
21–25	3 (20.0)
26–30	2 (13.3)
31–35	4 (26.7)
36–40	2 (13.3)
≥ 41	1 (6.7)
**Educational attainment**	
High school or GED	3 (20.0)
Technical/trade school, some college or 2-year degree	5 (33.3)
4-year college graduate	3 (20.0)
Postgraduate degree	4 (26.7)
**Married or Living with Partner**	12 (80.0)
**Current WIC participant**	5 (33.3)
**Covered by TennCare**	6 (40.0)
**PA prior to pregnancy**^**a**^	
I did not do much physical activity. I mostly did things like watching TV or reading. Only occasionally, no more than once or twice a month, did I do anything more active like going on a walk	1 (6.7)
Once or twice a week, I did light activities such as getting outdoors on the weekends for an easy walk or stroll. Or once or twice a week, I did chores around the house such as vacuuming	5 (33.3)
About 3 times a week, I did moderate activities such as brisk walking or biking for about 15–20 min each time. Or about once a week, I played sports or did a dance class for about 45–60 min	5 (33.3)
About 5 or more times a week, I did moderate activities such as brisk walking or biking for 30 min or more each time. Or about once a week, I played sports or did a dance class for about 2 h	2 (13.3)
About 3 times a week, I did vigorous activities such as running or riding hard on a bike for 30 min or more each time	2 (13.3)
About 5 or more times a week, I did vigorous activities such as running or riding hard on a bike for 30 min or more each time	0
**PA in the last 30 days**^**b**^	
I did not do much physical activity. I mostly did things like watching TV or reading. Only occasionally, no more than once or twice a month, did I do anything more active like going on a walk	2 (13.3)
Once or twice a week, I did light activities such as getting outdoors on the weekends for an easy walk or stroll. Or once or twice a week, I did chores around the house such as vacuuming	8 (53.3)
About 3 times a week, I did moderate activities such as brisk walking or biking for about 15–20 min each time. Or about once a week, I played sports or did a dance class for about 45–60 min	4 (26.7)
About 5 or more times a week, I did moderate activities such as brisk walking or biking for 30 min or more each time. Or about once a week, I played sports or did a dance class for about 2 h	1 (6.7)
About 3 times a week, I did vigorous activities such as running or riding hard on a bike for 30 min or more each time	0
About 5 or more times a week, I did vigorous activities such as running or riding hard on a bike for 30 min or more each time	0

*BMI* Body mass index, *GED* General Educational Development test or General Educational Diploma, *PA* Physical activity, *TennCare* The state of Tennessee’s Medicaid program, *WIC* Special Supplemental Nutrition Program for Women, Infants, and Children

^a^Before this pregnancy, which statement best describes the kinds of PA you usually did? Do not include the time you spent working at a job

^b^During the last 30 days, which statement best describes the kinds of PA you usually did? Do not include the time you spent working at a job

During the 200-m walk, participants (*n* = 10) walked at a mean cadence of 108.5 steps per minute (SD 6.5) by hand tally, and 100.7 steps per minute (SD 5.5) by the FC3 (Table 2). The mean pace was 68.2 m per minute (SD 10.7), or 2.5 miles per hour. During the metronome-assisted bouts, mean actual cadence (i.e., by hand-tally) most closely aligned with the prescribed cadence for the stepping-in-place bouts (Table 2). The results of the equivalence tests are also presented in Table 2. During the self-paced 200 m walk, the FC3 was equivalent (within 15%) to the hand-tallied criterion. In the metronome-assisted bouts, FC3 step counts were equivalent to hand-tallied counts for walking and stepping-in-place at 84 and 100 steps per minute with alpha at 0.05; walking at 100 steps per minute was no longer equivalent upon Bonferroni adjustment (i.e., alpha of 0.007). FC3 step counts for both walking and stepping-in-place at 64 steps per minute were non-equivalent to hand-tallied step counts.

**Table 2:** Mean cadences and step counts, equivalence testing, the percentage of steps accurately recorded, and mean absolute percent error for the Fitbit Charge 3 versus hand-tally

	**Mean FC3 Cadence**^**a**^	**Mean Hand-Tallied Cadence**^**b**^	**Mean FC3 Step Count**^**a**^	**Mean Hand- Tallied Step Count**^**b**^	***P***^**c**^	**Percentage of Steps Accurately Recorded**^**d**^	**Mean Absolute Percent Error**^**e**^
	**Steps per Minute (SD)**	**Steps per Minute (SD)**	**Steps (SD)**	**Steps (SD)**		**% (SD)**	**% (SD)**
**Self-paced 200 m Walk** (*n* = 10)	100.7 (5.5)	108.5 (6.5)	300.3 (36.5)	323.2 (37.2)	< .001	92.9 (2.8)	7.1 (2.8)
**Metronome-Assisted Bouts** (*N* = 15)							
Walking at 67 steps per minute	82.8 (7.8)	73.7 (9.0)	165.7 (15.6)	147.5 (17.9)	0.24	113.4 (14.2)	16.0 (11.0)
Walking at 84 steps per minute	86.8 (6.3)	90.5 (7.9)	173.5 (12.6)	180.9 (15.7)	< .001	96.1 (2.9)	4.2 (2.5)
Walking at 100 steps per minutes	94.5 (10.6)	102.0 (6.5)	189.1 (21.2)	203.9 (12.9)	0.01	93.0 (11.0)	7.0 (11.0)
Stepping at 67 steps per minute	76.1 (21.7)	67.8 (3.3)	152.2 (43.3)	135.5 (6.7)	0.38	113.2 (35.3)	30.4 (21.0)
Stepping at 84 steps per minute	85.3 (16.6)	86.6 (4.3)	170.6 (33.3)	173.2 (8.7)	0.006	98.4 (18.3)	12.6 (13.0)
Stepping at 100 steps per minutes	94.5 (8.9)	98.2 (5.9)	189.1 (17.8)	196.4 (11.9)	< .001	96.3 (7.7)	5.5 (6.4)

^a^Mean Fitbit Charge 3 cadence and step count across all participants

^b^Mean hand tally cadence and step count across all participants; each participant’s individual value is the mean of the two step counts obtained from research assistant step counters

^c^Equivalence testing by the two one-sided tests (TOST) method for ± 15% equivalence region

^d^Percentage of Steps Accurately Recorded = Σ ((Fitbit Charge 3 step count)/(hand-tallied step count) × 100)

^e^Mean Absolute Percent Error (MAPE) = Σ (|(hand-tallied step count—Fitbit Charge 3 step count)/(hand-tallied step count)|× 100)

**Fig. 2:**
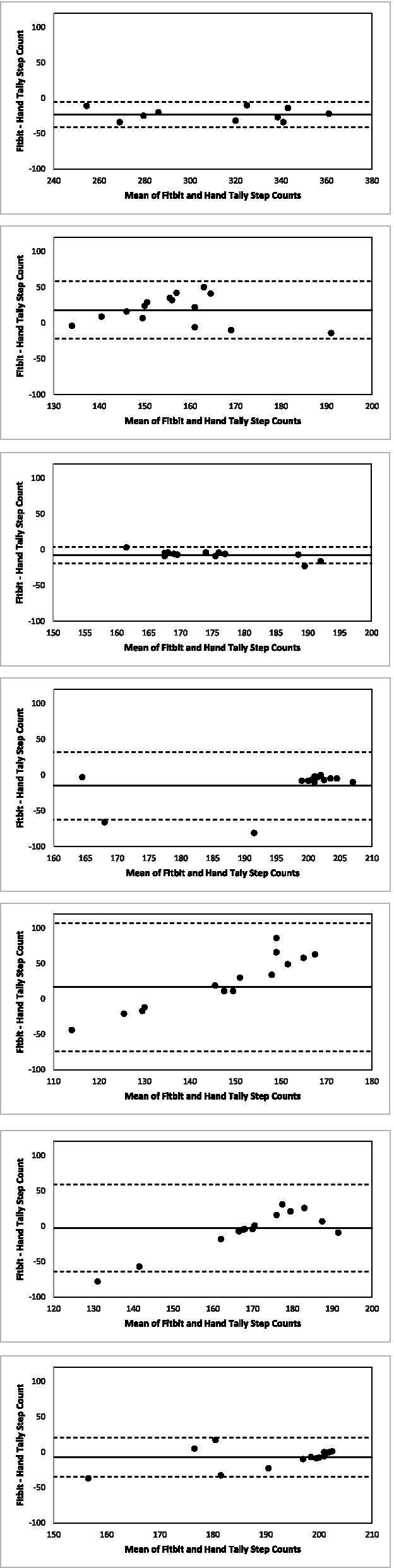
Bland Altman plots for the self-paced 200-m walk and the metronome-assisted bouts of walking and stepping-in-place

Figure 2 displays Bland Altman plots for the step count data presented in Table 2, and the parameters of the plots are presented in Table 3. The differences did not follow a normal distribution for walking at 84 and 100 steps per minute and stepping at 100 steps per minute according to Kolmogorov–Smirnov tests (Table 3). Visual assessment of the histograms of the differences confirmed departures from normal for walking and stepping at 100 steps per minute, both due to a high proportion of zero difference for those trials. Visual assessment of the Bland Altman plots revealed potential heterogeneity of the variances across the measurement range for several bouts (Fig. 2). The FC3 overestimated steps at a cadence of 64 steps per minute (i.e., positive bias), and slightly underestimated steps for 84 and 100 steps per minute and for the 200-m self-paced walk (i.e., negative bias). Limits of agreement for the individual bouts were wide, particularly for walking and stepping at the slowest cadence (Table 3 and Fig. 2). Relative to mean hand-tallied step count, the bias was greatest in magnitude at the slowest cadence, but was < 10% for walking and stepping at 84 and 100 steps per minute and during the self-paced walk. Walking and stepping at 100 steps per minute, walking at 84 steps per minutes, and the self-paced 200-m walk also had MAPEs within 10% (i.e., the accuracy standard of the Consumer Technology Association (CTA)™ [24], Table 2).

**Table 3:** Parameters of the Bland–Altman plots

	**Mean Hand- Tallied Steps (SD)**	**Kolmogorov–Smirnov test for Normality**	**Bias**		**Limits of Agreement**
			**Mean of the Differences**	**95% Confidence Interval**	
**Self-paced 200 m Walk** (*n* = 10)	323.2 (37.2)	> 0.150	-22.9	(-28.2, -17.6)	(-40.8, -5.0)
**Metronome-Assisted Bouts** (*N* = 15)					
Walking at 67 steps per minute	147.5 (17.9)	> 0.150	18.2	(8.9, 27.5)	(-21.9, 58.3)
Walking at 84 steps per minute	180.9 (15.7)	< 0.010^a^	-7.4	(-10.1, -4.7)	(-18.9, 4.1)
Walking at 100 steps per minutes	203.9 (12.9)	< 0.010^a^	-14.9	(-25.8, -3.9)	(-62.2, 32.5)
Stepping at 67 steps per minute	135.5 (6.7)	> 0.150	16.7	(-4.3, 37.6)	(-73.6, 106.9)
Stepping at 84 steps per minute	173.2 (8.7)	0.05	-2.6	(-16.9, 11.7)	(-64.2, 59.0)
Stepping at 100 steps per minutes	196.4 (11.9)	0.039^a^	-7.3	(-13.8, -0.9)	(-35.2, 20.5)

^a^Differences do not follow a normal distribution

The semi-structured interviews revealed that participants were willing to engage in PA for health (i.e., their own health and that of the baby). *“It would be healthier for me and the baby.”* However, there were many barriers cited preventing engagement in PA, including other children or family members to care for, full/part time jobs, and the physical discomforts of pregnancy, e.g., *“it’s just having a toddler that makes things a little bit more difficult.”* Convenience was a recurring theme: simplifying PA or integrating activities into a typical day would be preferred, e.g., “*it’d probably be on my break just like keep walking in place or something.”* Participants relayed their willingness to walk and for stepping-in-place as an alternative when unable to walk, and were open to the idea of using a FC3 to record episodes of walking and stepping. Two participants stated, *“Thirty minutes that you could split up, for sure. Especially if it [FC3] keeps track of what you do that day.”* Some participants stated that they were already active due to their many daily responsibilities and busy lives, but described participation in activities associated with daily living only (as opposed to activity that is done intentionally for health or wellness). For example, “*I live in a house full of people so I get up and I’ll sweep and mop and I do laundry, do grocery shopping. I’m always out shopping so I get of lot of walking and exercise.”*

## Discussion

The findings of the present study suggest that women with GDM face challenges finding time for PA given their busy schedules and competing demands. However, participants seemed open to the idea of increasing PA by walking and stepping-in-place, and to using a FC3 to record and monitor their walking and stepping. The FC3 performed adequately when walking or stepping-in-place at cadences of approximately 84 and 100 steps per minute, the latter just under their preferred walking cadence during the 200-m walk. Importantly, the FC3 met the accuracy standard of the Consumer Technology Association (CTA)™ during walking at 84 steps per minutes, walking and stepping at 100 steps per minute, and during the 200-m self-paced walk [24].

The FC3 overestimated steps when walking or stepping at the lowest cadence examined (i.e., 67 steps per minute). This is in contrast to previous studies in pregnant women, which report that devices under-estimate step counts at slower walking speeds [27]. The 67 steps per minute cadence felt unnaturally slow to many participants, and likely led to excess movement of the upper extremities for several participants when they got off cadence. Overall, the present study’s findings suggest that the FC3 is acceptable for counting steps while walking and stepping in late pregnancy, particularly when at or close to women’s preferred cadence.

Previous successful lifestyle interventions for weight management (i.e., PA and diet) targeting pregnant and postpartum women [8–10] set in the health care system utilize behavior change techniques [28] including, but not limited to, self-monitoring of and feedback on behavior (e.g., daily self-monitoring of minutes walking or number of steps); goal setting; review of behavior goals (e.g., reviewing behavior logs and modifying behavior goals accordingly); and problem solving (e.g., when to fit walking or stepping into a typical day). Components of these behavior change techniques are already incorporated into standard clinical care for women with GDM, for example, the provision of an individualized daily carbohydrate gram goal (i.e., medical nutrition therapy) and/or caloric intake goal (i.e., weight management), reviewing behavior progress with a diabetes educator, and modifying the goals accordingly [3, 4]. Women with GDM could be given PA goals that include a step goal to promote moderate intensity walking and/or stepping-in-place. Studies of pregnant [29] and non-pregnant adults [21, 30] identify 100 steps per minute as indicative of moderate intensity walking. A small study of non-pregnant adults found that stepping-in-place had a similar caloric requirement to walking at 3.0 mph on a treadmill [14], but the cadence while stepping-in-place was not reported.

A small pilot study of a clinic-based intervention in 17 women with type 2 or gestational diabetes, who were recruited in the second trimester, included one-on-one counseling and step goal setting; counseling sessions occurred in the clinic, immediately following a prenatal care visit [7]. Participants were given a Fitbit Alta HR monitor and shown how to use the app. In the 12-week intervention, participants were given individualized step goals and encouraged to work up to 10,000 steps per day. Adherence to wearing the Fitbit was relatively high and the intervention generally well accepted, with 85% indicating that the Fitbit helped them to increase PA. Daily steps increased from baseline (mean 6122, SD 2439) to the third week of the intervention (mean 6269, SD 2166), and then decreased through the 12^th^ week of the intervention (mean 4191, SD 2228). It is worth noting that PA decreases as pregnancy progresses [31], thus the lack of a concurrent control arm complicates interpretation of the last finding (e.g., it remains unknown whether the intervention resulted in less of a decrease in PA through the third trimester) [7].

There are few published data on stepping-in-place overall. One small randomized controlled trial examined stepping-in-place or walking around the house while watching TV commercials (i.e., during at least 90 min of TV programming, ≥ 5 days/week) as convenient mode of moderate-intensity activity to replace (i.e., reduce) sedentary time [14]. Although TV commercials are now less pervasive, a ‘lack of time’ participate in PA and issues pertaining to convenience continue to be barriers. Women with GDM may find it easier to increase their PA by intentionally stepping-in-place or around the house more, while talking on the phone, tidying the house or performing other activities that are already a part of their everyday routine.

Although steps obtained from walking differ from those obtained while stepping-in-place, the behavioral intervention component of problem solving makes stepping-in-place attractive for this population. It can be done anywhere and simultaneously with other daily activities. However, women’s satisfaction with and enjoyment from this mode of activity, important constructs in terms of motivation for PA [32], remain to be determined. Important for the behavioral intervention component of tracking and feedback towards a step goal, the FC3’s step count for both walking and stepping-in-place at 84 and 100 steps per minute were adequately aligned with hand-tallied step counts. These cadences are lower than the natural walking cadence observed during the self-paced walk in the present study (i.e., 68.2 m per minute) and a previous study (i.e., 68.5 m per minute walking a 400-m track) [20]. However, to meet recommendations for PA, women would need to walk or step for a significantly longer duration than examined in the present study. It is thus reasonable to suggest that for some portion of their walking/stepping, the cadence would be slower than the natural walking cadence observed in the present study. Data from ActiGraph accelerometers worn on the hip during the third trimester of pregnancy suggest that at this point in pregnancy, women spend only 0.82% of their waking hours at cadences ≥ 100 steps per minute [22].

There are other limitations worth noting. Findings from this small sample may not be generalizable, particularly the qualitative findings, given the participation rate. Finally, while consideration of MAPE is recommended because it reflects error expected at the individual level [23], the present study’s protocol differed from the CTA Standard’s mandatory testing conditions, under which the 10% MAPE cutoff is meant to be applied. Several of the Bland Altman plots should be interpreted with caution (e.g., for the distribution of the differences departing from normal and/or lack of homogeneity of variance). However, the Bland Altman plot for the 200 m self-paced walk met both of these assumptions and suggested a slight bias (i.e., underestimation of the step count) at the women’s preferred pace, though the limits of agreement were wide, suggesting room for improvement in terms of precision. Taken together with the results of the equivalence testing, the FC3’s step count appears to be adequate at women’s preferred pace and overall performs better at faster speeds or cadences.

## Conclusions

Results of the present study suggest that women with GDM are willing and motivated to participate in PA for their health and that of the baby, though it needs to be simple and convenient. Walking and stepping-in-place were acceptable modes of PA, and suitable given the commonly stated barrier that it was difficult to fit PA into their busy lifestyles. Future studies are needed to understand adherence to, acceptance of and satisfaction with stepping-in-place for PA. Results also suggest that the FC3, a commercial step counter, is adequate for counting steps while walking and stepping-in-place during the third trimester of pregnancy, particularly when walking and stepping at or close to their preferred cadence.

## Data Availability

Study data are not available due the small size of the sample and concern that individual privacy could be compromised but are available from the corresponding author on reasonable request.
